# Lateral Mobility and Nanoscale Spatial Arrangement of Chemokine-activated α4β1 Integrins on T Cells*[Fn FN2]

**DOI:** 10.1074/jbc.M116.733709

**Published:** 2016-08-01

**Authors:** Alberto Sosa-Costa, Sol Isern de Val, Silvia Sevilla-Movilla, Kyra J. E. Borgman, Carlo Manzo, Joaquin Teixidó, Maria F. Garcia-Parajo

**Affiliations:** From the ‡ Institut de Ciencies Fotoniques, Barcelona Institute of Science and Technology, 08860 Castelldefels, Barcelona, Spain,; the § Centro de Investigaciones Biológicas, Department of Cellular and Molecular Medicine, 28040 Madrid, Spain, and; the ¶ ICREA, Pg. Lluís Companys 23, 08010 Barcelona, Spain

**Keywords:** cell adhesion, cell migration, integrin, membrane biophysics, microscopy, single particle analysis

## Abstract

Chemokine stimulation of integrin α4β1-dependent T lymphocyte adhesion is a key step during lymphocyte trafficking. A central question regarding α4β1 function is how its lateral mobility and organization influence its affinity and avidity following cell stimulation with chemokines and/or ligands. Using single particle tracking and superresolution imaging approaches, we explored the lateral mobility and spatial arrangement of individual α4β1integrins on T cells exposed to different activating stimuli. We show that CXCL12 stimulation leads to rapid and transient α4β1activation, measured by induction of the activation epitope recognized by the HUTS-21 anti-β1antibody and by increased talin-β1 association. CXCL12-dependent α4β1 activation directly correlated with restricted lateral diffusion and integrin immobilization. Moreover, co-stimulation by CXCL12 together with soluble VCAM-1 potentiated integrin immobilization with a 5-fold increase in immobile integrins compared with unstimulated conditions. Our data indicate that docking by talin of the chemokine-activated α4β1 to the actin cytoskeleton favors integrin immobilization, which likely facilitates ligand interaction and increased adhesiveness. Superresolution imaging showed that the nanoscale organization of high-affinity α4β1 remains unaffected following chemokine and/or ligand addition. Instead, newly activated α4β1 integrins organize on the cell membrane as independent units without joining pre-established integrin sites to contribute to cluster formation. Altogether, our results provide a rationale to understand how the spatiotemporal organization of activated α4β1 integrins regulates T lymphocyte adhesion.

## Introduction

Integrins are heterodimeric cell membrane adhesion receptors composed of non-covalently associated α and β subunits that mediate cell-cell and cell-extracellular matrix adhesion (1). Integrins control morphogenesis, immunity, tissue healing, and tumor growth and metastasis. In particular, the α4β1 (VLA-4) and αLβ2 (LFA-1) integrins are key players in T lymphocyte trafficking from the blood circulation to lymphoid tissues and to sites of injury and infection (2). Their adhesiveness is rapidly and transiently activated by chemokines (3, 4), allowing highly dynamic T cell interactions with the endothelium that facilitate crawling and diapedesis. Binding of chemokines to their receptors generates an inside-out signaling that impinges on the cytoplasmic domains of β subunits (5–7), leading to the extension of high-affinity conformations of the extracellular α4β1 and αLβ2 integrins that are competent for binding to their ligands VCAM-1 and ICAM-1, respectively. It is generally accepted that interactions of talin and kindlin with specific cytoplasmic motifs on β1 and β2 integrin subunits represent critical steps for the generation of active α4β1 and αLβ2 integrins (7–10).

The role played by α4β1 is especially important during T lymphocyte trafficking to sites of inflammation. Its interaction with VCAM-1 as well as with the CS-1 region of fibronectin allows optimal T cell migration, which contributes to subsequent immune responses. In addition to the critical involvement of talin in stimulating active α4β1 conformations (11, 12), the characterization of the inside-out signaling required for chemokine-promoted α4β1 activation revealed that Vav1 plays a key role in the optimal stimulation of T cell adhesion mediated by this integrin (13).

A central question regarding integrin function on lymphocytes is how the lateral organization and mobility of α4β1 and αLβ2 influence their activation and adhesiveness after contact with chemokines and/or ligands. This question is important to improve our understanding on how these integrins spatially regulate their affinity and avidity and would ultimately allow interference in this process. Earlier reports showed that lateral mobility controls αLβ2 rearrangement into clusters upon leukocyte activation, which occurs following release of cytoskeleton constraints, allowing integrin motion (14). These studies led to the proposal that inactive αLβ2 integrins are anchored to the cytoskeleton and released to strengthen ligand binding (14), suggesting that αLβ2 integrin activation precedes clustering. However, high-affinity integrins are more prone to interact with the cytoskeleton via their cytoplasmic domains (1), causing integrin immobilization and compromising their lateral diffusion. Indeed, high-affinity αLβ2 appears immobile in phorbol 12-myristate 13-acetate-activated cells (15). Along the same lines, we recently showed that αLβ2 activation by extracellular cations or chemokine stimulation increased the percentage of immobile αLβ2 nanoclusters in dendritic cells, indicating that αLβ2 immobilization correlates with integrin activation (16, 17).

Little is known about the mobility of α4β1integrins on lymphocytes. On BMPCs,^4^ the lateral diffusion of α4 integrins was found to be slow and correlated with strong BMPC adhesiveness (18). Furthermore, examination of the lateral diffusion of a mutant *Drosophila* αPS2βPS integrin exhibiting high affinity for its ligand revealed slower diffusion than the wild-type counterpart (19). No studies have yet been undertaken that focus on the membrane lateral organization of α4β1 following lymphocyte exposure to chemokines and/or ligands. Here we applied single-molecule approaches and superresolution microscopy together with reporters of β1 activation to study the potential lateral mobility alterations and spatial regulation of α4β1 in response to chemokine and/or ligand stimuli.

## Results

### 

#### 

##### Chemokine Stimulation Transiently Restricts the Lateral Mobility of α4β1 Integrins on T Cells

**FIGURE 1.**
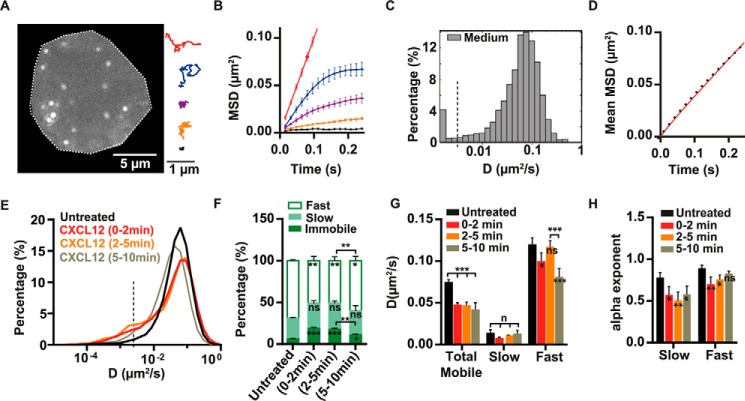
**Characterization of the lateral mobility of α4β1 on T cells and effect of CXCL12 stimulation.** *A*, representative frame from the tracking of individual α4β1 molecules on Molt-4 T cells using clone 18 anti-β1-QD-655 conjugates (an untreated cell is depicted). Examples of individual α4β1 trajectories displaying different types of motion are shown in the *right panel. B*, MSD over time for representative trajectories displayed in *A* using the same color code. *C*, semi-log histogram of D_1–4_ for α4β1. Values below D_1–4_ = 0.0025 μm^2^/s (*dashed vertical line*) correspond to immobile trajectories. *D*, overall MSD plot of the total mobile α4β1 population at different time lags. *E*, overlay semi-log distributions of D_1–4_ for the indicated incubation conditions. The das*hed vertical line* at D = 0.0025 μm^2^/s denotes the threshold value for discriminating immobile (*left*) from mobile (*right*) trajectories. *F*, percentage of immobile, slow, and fast α4β1 subpopulations for untreated or CXCL12-exposed cells. *G*, diffusion coefficient values of the total mobile population and for the slow and fast α4β1 fractions. *H*, anomalous α exponents for the slow and fast α4β1 subpopulations under the indicated conditions. Unless indicated otherwise, statistical comparisons were done with respect to untreated cells. Statistics over three separate experiments are as follows: 4021 trajectories on 40 cells (untreated) (*C* and *D*); for different conditions (*E–H*), 20 untreated cells (1637 trajectories), 6 cells stimulated with CXCL12 for 0–2 min (816 trajectories), 9 cells stimulated with CXCL12 for 2–5 min (1014 trajectories), and 21 cells stimulated with CXCL12 for 5–10 min (1937 trajectories). *ns*, not significant (*p* > 0.05); *, *p* < 0.05; **, *p* < 0.01; ***, *p* < 0.001.

The chemokine CXCL12 triggers an inside-out signaling that induces high-affinity conformations of α4β1, leading to strengthening of α4β1-VCAM-1 interaction and to increased leukocyte adhesiveness (13). To investigate the effect of chemokine stimulation on α4β1 lateral mobility on T cells, we used SPT approaches (20). Molt-4 cells were employed as a model, as α4β1 constitutes the predominant β1 integrin heterodimer in these cells, with very low α5β1 expression (supplemental Fig. S1), and it is highly responsive to CXCL12 stimulation (13). Cells were stretched onto PLL-coated coverslips and labeled at low density with the conformation-independent anti-β1 clone 18 antibody previously biotinylated and conjugated with streptavidin-coated QD655. To ensure a 1:1 QD:antibody stoichiometry, the anti-β1-QD conjugate was prepared in an excess of free biotin to occlude streptavidin-QD extra binding sites. We recorded the motion of individual QDs by using an SPT setup working under oblique illumination. Subsequently, trajectories were reconstructed and analyzed. To minimize effects of internalization of the conjugated antibodies, measurements were always performed during the first 20 min after labeling. Moreover, to prevent potential artifacts because of the relative large size of QDs and the proximity between the cell membrane and the substrate, we exclusively imaged the apical side of the cells (Fig. 1*A*, *left panel*). Experiments were carried out as follows. During the first 10 min, measurements of α4β1 integrin diffusion were performed with T cells kept in RPMI 1640 medium, *i.e.* the untreated condition. Then CXCL12 was added and maintained for another 10 min. Measurements during this period were further separated into three time windows: 0–2 min, 2–5 min, and 5–10 min.

In general, the trajectories of individual α4β1 integrins under untreated conditions exhibited heterogeneous behavior, with some trajectories showing high mobility and others a more restricted motion (Fig. 1*A*, *right panel*, and supplemental Movie S1). To quantitatively describe the diffusion of α4β1, MSD curves were generated per trajectory (examples are shown in Fig. 1*B*). Different diffusion modes were detected: immobile, confined, and Brownian motion (Fig. 1, *A* and *B*). The diffusion coefficients at short time lags (D_1–4_) were then determined by linear fitting of the MSD curves over the first four points, and their values were plotted as a semi-log histogram to show the full distribution of the α4β1 diffusive behavior. On untreated cells, α4β1 integrins showed a broad variation over more than 2 orders of magnitude in the D_1–4_ values, with only a small fraction being immobile (∼6%; see “Experimental Procedures”) (Fig. 1*C*). On the other hand, the mean MSD curve of the total mobile population showed a linear relationship with time lag (Fig. 1*D*), indicating Brownian diffusion of the integrin on unperturbed cells, with a mean value of D = (0.083 ± 0.005) μm^2^/s.

Addition of CXCL12 significantly altered α4β1 diffusion during the first 2 min after stimulation, with the appearance of a shoulder at lower D_1–4_ values and an increase of immobile trajectories compared with untreated samples (Fig. 1*E*). To better quantify these changes, we applied CPD analysis on the mobile α4β1 population (21). By using this approach, two different α4β1 subpopulations having slow and fast diffusion were retrieved (see “Experimental Procedures”). For each subpopulation we determined their relative occurrence (expressed in percentages), average diffusion coefficient D*_i_*, and anomalous α*_i_* exponents (where α indicates the type of motion, *i.e.* α = 1 for Brownian diffusion and α < 1 for anomalous diffusion) with the subscript *i* = *s,f* referring to the slow or the fast subpopulation, respectively.

A remarkable 3-fold increase in immobile α4β1 trajectories (from 5% to 20%) was observed during the first 2 min of CXCL12 treatment compared with untreated cells (Fig. 1*F* and supplemental Fig. S2*A*). This increase was mostly due to a reduction of the fast subpopulation (from 70% to 50%) (Fig. 1*F*). In addition, the D values for the total mobile and the fast subpopulation were reduced with respect to unstimulated cells (Fig. 1*G*) together with a slight but significant increase in the anomalous diffusion of the fast subpopulation (from α*_f_* = 0.89 to α*_f_* = 0.76; Fig. 1*H*). Moreover, although the percentage and diffusion coefficient of the slow mobile subpopulation did not significantly change upon CXCL12 stimulation (Fig. 1, *F* and *G*), its mobility became highly anomalous (from α*_s_* = 0.78 to α*_s_* = 0.48) (Fig. 1*H*). These major changes in α4β1 mobility were maintained during the subsequent 2–5 min of CXCL12 exposure (Fig. 1, *E–H*).

During the 5- to 10-min time window after CXCL12 addition, the overall distribution of D_1–4_ remained shifted toward lower values with respect to untreated cells (Fig. 1*E*), with comparable values in terms of diffusion coefficients (Fig. 1*G*) and anomalous behavior (Fig. 1*H*) to those obtained during the first 5 min of CXCL12 stimulation. However, a partial recovery of the percentage of mobile molecules was observed in the 5- to 10-min period of CXCL12 treatment, concomitant with a decrease in the percentage of immobile α4β1 (around 11%) compared with the first 5-min interval (Fig. 1*F* and supplemental Fig. S2*A*). Together, these results show that CXCL12 stimulation leads to both immobilization and slowing down of the overall lateral mobility of α4β1 integrins on the surface of T cells. Because CXCL12 promotes high-affinity α4β1, these results suggest the existence of a direct correlation between the integrin-restricted diffusion and increased immobilization and its activation. Moreover, the recovery in the mobile fraction in the 5- to 10-min period of exposure to CXCL12 is consistent with the reported transient effect of chemokines on integrin activation (14, 17, 22–25).

##### α4β1 Integrin Immobilization Correlates with Integrin Activation

**FIGURE 2.**
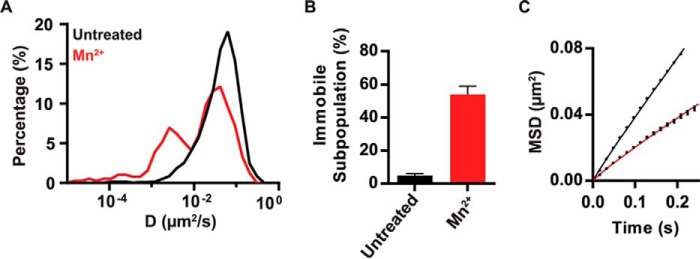
**Effect of Mn^2+^ stimulation on the lateral mobility of α4β1 on T cells.** *A*, overlay semi-log distributions of D_1–4_ values for untreated Molt-4 cells (*black line*) or stimulated with Mn^2+^ for 10 min (*red line*). *B*, plot of the immobile trajectories for both conditions. *C*, MSD plot of mobile trajectories as a function of time for untreated (*black line*) and Mn^2+^-treated cells (*red line*). Seven untreated cells (320 trajectories) and eight cells stimulated with Mn^2+^ (223 trajectories) over two separate experiments.

To determine whether the level of integrin activation is indeed linked to α4β1 immobilization, we exposed Molt-4 cells to Mn^2+^, a potent extracellular activator of integrin affinity (26). Activated α4β1 showed a high percentage of immobile integrins compared with untreated cells (Fig. 2, *A* and *B*) and nearly the same slower diffusion as that obtained upon CXCL2 treatment, with mean D values of 0.040 ± 0.004 μm^2^/s and D = 0.043 ± 0.009 μm^2^/s for Mn^2+^ and CXCL2 treatment, respectively (Fig. 2*C*). These results thus confirm that immobilization and reduced mobility of α4β1 directly correlate with integrin activation.

##### Soluble VCAM-1 Has a Weak Effect on the Lateral Diffusion of α4β1 Integrins

**FIGURE 3.**
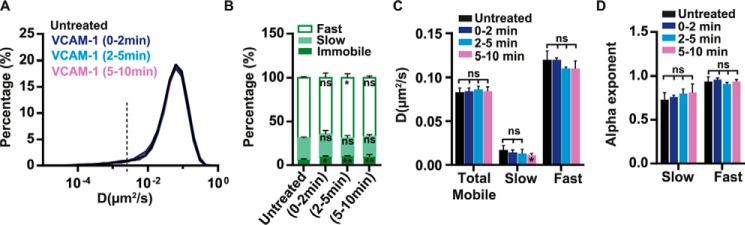
**Effect of soluble VCAM-1 on the lateral mobility of α4β1in T cells.** *A*, overlay semi-log distributions of D_1–4_ values for untreated cells or cells exposed to soluble VCAM-1-Fc in solution for 0–2, 2–5, and 5–10 min. *B*, percentage of immobile, slow, and fast α4β1 subpopulations for untreated or VCAM-1-exposed cells. *C*, diffusion coefficients of the total mobile population and for the slow and fast fractions of α4β1. *D*, anomalous α exponents for the slow and fast α4β1 subpopulations. Ten untreated cells (1939 trajectories), five cells stimulated with VCAM-1 for 0–2 min (787 trajectories), eight cells stimulated with VCAM-1 for 2–5 min (1582 trajectories), and 11 cells stimulated with VCAM-1 for 5–10 min (1550 trajectories) over three separate experiments. *ns*, not significant (*p* > 0.05); *, *p* < 0.05; **, *p* < 0.01.

Because ligands can activate integrins extracellularly (1, 27), we sought to investigate whether Molt-4 exposure to soluble VCAM-1-Fc would also influence the lateral mobility of α4β1. Surprisingly, no differences in the diffusivity of α4β1 molecules were observed after ligand addition (Fig. 3, *A–D*). However, a nearly 2-fold increase in the percentage of immobile trajectories, from 6% (untreated) to 11% upon VCAM-1 stimulation, was observed during the first 2 min of VCAM-1 exposure (supplemental Fig. S2*B*), and this percentage remained constant up to 10 min (our observation time) (Fig. 3*B* and supplemental Fig. S2*B*). Therefore, these data indicate that soluble VCAM-1-Fc is capable of inducing stable immobilization of a small subset of integrins without altering the overall diffusion of the remaining mobile molecules.

##### Addition of Soluble VCAM-1 to CXCL12 Stimulation Increases Immobilization of α4β1 Integrins

**FIGURE 4.**
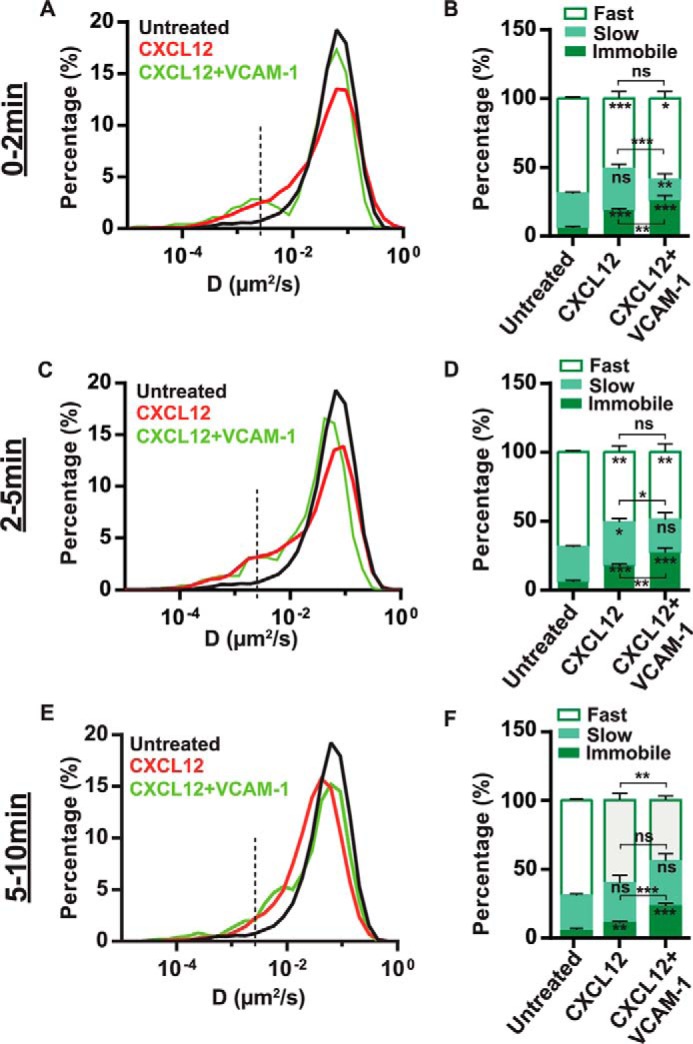
**Effect of CXCL12 and VCAM-1 co-stimulation on the lateral mobility of α4β1in T-cells.** *A* and *B*, data correspond to the first 0- to 2-min period of the different stimulation conditions. *A*, overlay semi-log distributions of D_1–4_ values for the indicated conditions. Data from the CXCL12 stimulation experiments (Fig. 1) are included to allow easier comparison of both conditions. *B*, percentage of immobile, slow, and fast α4β1 subpopulations. *C* and *D*, the same as *A* and *B* at the 2- to 5-min interval of stimulation. *E* and *F*, the same as in *A* and *B* at the 5- to 10-min interval of stimulation. 29 untreated cells (2382 trajectories), five cells co-stimulated with CXCL12 and VCAM-1 (260 trajectories) during 0–2 min, seven cells co-stimulated with CXCL12 and VCAM-1 (301 trajectories) during 2–5 min, and nine cells (587 trajectories) during 5–10 min over two separate experiments. *ns*, not significant (*p* > 0.05); *, *p* < 0.05; **, *p* < 0.01; ***, *p* < 0.001.

Given the strong relationship between CXCL12-induced α4β1 integrin activation and its restricted diffusion, we reasoned that the minor changes in α4β1 mobility observed after exposure to soluble VCAM-1 could indicate that the ligand alone is insufficient to fully activate the integrin. We thus inquired whether co-stimulation by both CXCL12 and soluble VCAM-1 could alter α4β1 diffusivity beyond that of VCAM-1 or CXCL12 alone. During the first 0- to 2-min treatment with CXCL12 and VCAM-1-Fc, we detected a prominent shoulder at lower D_1–4_ values compared with unstimulated cells or those stimulated with VCAM-1-Fc or CXCL12 alone (Fig. 4*A*). Importantly, combined stimulation by CXCL12 and VCAM-1 resulted in a remarkable increase in α4β1 immobilization (∼30% *versus* 6% for untreated cells and 20% for CXCL12 alone, Fig. 4*B* and supplemental Fig. S2*C*) that was maintained during the subsequent 2–5 min of combined stimulation (Fig. 4, *C* and *D*, and supplemental Fig. S2*D*). Notably, this increase in the percentage of immobile integrins was mostly due to a reduction of the slow subpopulation (Fig. 4, *B* and *D*). On the other hand, the diffusion of the slow and fast mobile α4β1 subpopulations did not significantly change upon co-stimulation compared with CXCL12 alone (supplemental Fig. S3, *A–D*), indicating that CXCL12 is the main stimulus impacting α4β1 mobility. A similar trend was observed during 5–10 min of co-stimulation with CXCL12 and VCAM-1-Fc (Fig. 4, *E* and *F*, and supplemental Figs. S2*E* and S3, *E* and *F*), albeit more modest. That is, the percentage of immobile integrins in the presence of both stimuli was reduced from ∼30% to 23% (Fig. 4*F* and supplemental Fig. S2*E*) compared with cells co-stimulated for 0–5 min, suggesting that, similar to CXCL12, the combined effect of CXCL12 and VCAM-1 is also transient. Taken together, these results suggest that integrin binding to its soluble ligand alone does not lead to robust integrin immobilization and restricted diffusion, a condition best achieved upon simultaneous stimulation with CXCL12 and VCAM-1.

##### Immobilized VCAM-1 Strongly Arrests the Mobility of α4β1 Integrins, an Effect That Is Potentiated by CXCL12 Stimulation

**FIGURE 5.**
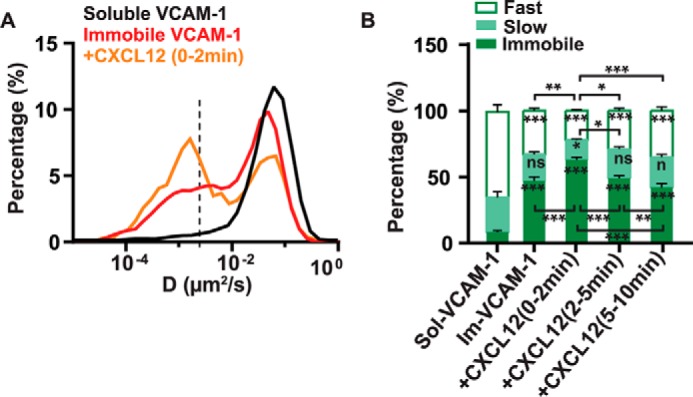
**Effect of immobilized VCAM-1 and CXCL12 co-stimulation on the lateral mobility of α4β1in T-cells.** *A*, overlay semi-log distributions of D_1–4_ values for soluble VCAM-1 (*black line*), immobilized VCAM-1 (*red line*), and immobilized VCAM-1 together with CXCL12 stimulation for 2 min (*orange line*). Data from the soluble VCAM-1 stimulation experiments (Fig. 3) are included to allow easier comparison of both conditions. *B*, percentage of immobile, slow, and fast α4β1 subpopulations for the different stimulation conditions compared with soluble VCAM-1 (*sol-VCAM-1*). *Im-VCAM-1*, immobilized VCAM-1. 18 untreated cells on immobilized VCAM-1 (1920 trajectories), seven cells stimulated with CXCL12 for 0–2 min (759 trajectories), eight cells stimulated with CXCL12 for 2–5 min (865 trajectories), and 13 cells stimulated with CXCL12 for 5–10 min (1588 trajectories) over three separate experiments. *ns*, not significant (*p* > 0.05); *, *p* < 0.05; **, *p* < 0.01; ***, *p* < 0.001.

It has been shown that multimeric and/or immobilized ICAM-1 ligands increase the high-affinity form of αLβ2 integrins (28) and strongly affect their lateral mobility on the cell surface (28, 17). To investigate the effect of immobilized *versus* soluble VCAM-1 on the diffusion profile of α4β1, we performed SPT experiments on Molt-4 cells seeded on immobilized VCAM-1. Immobilization of the ligand led to a massive reduction of α4β1 mobility (Fig. 5*A*) and more than a 4-fold increase in the percentage of immobilized integrins (Fig. 5*B*) compared with soluble ligand exposure (from 11% for soluble to 47% for immobilized VCAM-1). CXCL12 stimulation further increased α4β1 immobilization to 63% during the first 2 min of exposure (Fig. 5*B*). Again, the effect of CXCL12 was transient so that, after 2 min of chemokine stimulation, integrin immobilization progressively returned to similar values as those without stimulation. Interestingly, ligand immobilization also reduced the diffusion of mobile integrins to values comparable with those obtained upon CXCL12 and Mn^2+^ treatments (compare supplemental Fig. S4 with Figs. 1*G* and 2*C*). Thus, these results indicate that immobilized but not soluble VCAM-1 triggers the activation of α4β1 integrins, which is further potentiated by chemokine stimulation.

##### The Decrease in α4β1 Lateral Mobility by CXCL12 and CXCL12/VCAM-1 Is Linked to Integrin Activation

**FIGURE 6.**
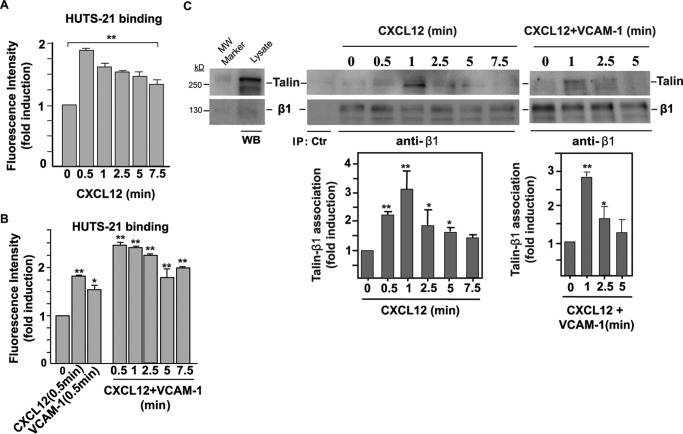
**Analyses of α4β1 integrin activation.** *A* and *B*, Molt-4 cells were exposed for the indicated times to CXCL12 or VCAM-1-Fc alone and subjected to flow cytometry with the HUTS-21 anti-β1 mAb (*n* = 3). *C*, cells were exposed to CXCL12 alone (*center panel*) or combined with soluble VCAM-1-Fc (*right panel*) for the indicated times and subsequently subjected to immunoprecipitation with the TS2/16 anti-β1 mAb followed by immunoblotting with antibodies to the indicated proteins. Also shown are densitometric analyses of gel bands from the immunoprecipitations displaying the mean ± S.D. of four independent experiments (*bottom panels*). *, *p* < 0.05; **, *p* < 0.01. *MW*, molecular weight; *WB*, Western blot; *IP*, immunoprecipitation.

The results shown so far indicate that α4β1 immobilization is increased in T cells exposed to different integrin-activating conditions. To further analyze whether this increase is directly linked to α4β1 activation, we followed the time-dependent expression of a β1 activation epitope on CXCL12-exposed Molt-4 cells by means of flow cytometry. As the activation reporter we used HUTS-21, an anti-β1 mAb that recognizes high-affinity β1 integrins (29). Because Molt-4 cells express predominantly α4β1 integrins (supplemental Fig. S1), HUTS-21 selectively reports on high-affinity α4β1. CXCL12 triggered a rapid induction of high-affinity α4β1 integrin conformations (Fig. 6*A*), with a strong reactivity to HUTS-21 already detected at 0.5 min of treatment. The induction of these high-affinity α4β1 forms decreased at longer times, although, after 7.5 min, their expression levels were still notably higher compared with untreated cells (Fig. 6*A*). VCAM-1-Fc alone was also able to induce high-affinity integrins at 0.5 min, albeit to a lower extent compared with CXCL12 (Fig. 6*B*). Importantly, co-stimulation by CXCL12 and VCAM-1-Fc resulted in a 2.5-fold increase in high-affinity integrins at 0.5 min that remained remarkably above that of untreated conditions even after 7.5 min of co-stimulation (Fig. 6*B*). Of note, the activation of α4β1 by CXCL12/VCAM-1 remained higher up to 2.5 min than that of single CXCL12 or VCAM-1 at the 0.5-min time point.

Next we assessed talin interaction with the β1 subunit as an additional measurement of integrin activation (30). For this purpose, we exposed Molt-4 cells for different times to CXCL12, and, following cell lysis, cell extracts were immunoprecipitated with the TS2/16 anti-β1 mAb and subjected to immunoblotting using anti-β1 and anti-talin antibodies (12). Similar to the results obtained by flow cytometry using the HUTS-21 mAb, the immunoprecipitation data indicated that β1-talin association was rapid and transient and mainly detected in the first 5 min of exposure to the chemokine, especially in the 0.5- to 1-min window, nicely coinciding (within the temporal accuracy of the experiments) with the reactivity to HUTS-21 (Fig. 6*C*, *center panel*). However, under our experimental immunoprecipitation conditions, we were not able to detect a further increase in β1-talin association upon addition of soluble VCAM-1 to CXCL12 compared with incubation with CXCL12 alone (Fig. 6*C*, *right panel*). Together, these results further confirm that the reduction in α4β1 mobility upon CXCL12 stimulation observed by SPT directly correlates with the activation of this integrin. Furthermore, as talin connects the β subunits with the actin cytoskeleton, our data strongly suggest that the observed reduction in α4β1 mobility is the result of its increased talin-dependent interaction with the cytoskeleton.

##### The Nanoscale Spatial Arrangement of α4β1 Integrins Is Unaffected by Integrin Activation

**FIGURE 7.**
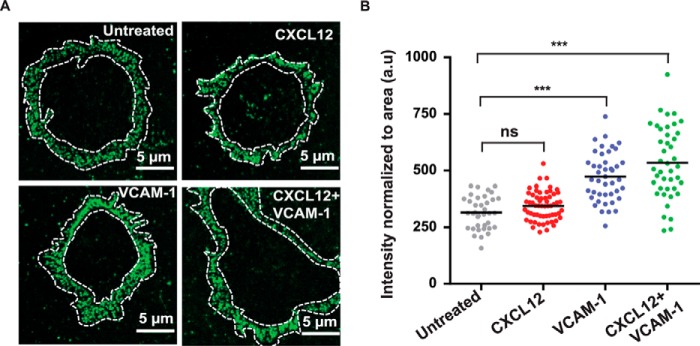
*A*, representative confocal images of Molt-4 cells labeled with HUTS-21 mAb for different stimulation conditions. The *white dashed lines* denote the regions of the cell membrane subjected to fluorescence intensity analysis. *B*, fluorescence intensity of HUTS-21 mAb normalized to the area for different conditions from confocal images of individual cells. Each dot corresponds to an individual cell. Unless indicated, statistical comparisons were done with respect to untreated cells. *ns*, not significant (*p* > 0.05); ***, *p* < 0.001; *a.u.*, arbitrary units.

To investigate the effect of integrin-activating conditions on the spatial regulation of α4β1, we performed high-resolution confocal and STED superresolution microscopy on Molt-4 cells. Cells were adhered onto PLL-coated slides and subjected to stimulation for 2.5 min by CXCL12, VCAM-1-Fc, or a combination of both, followed by rapid fixation and staining using HUTS-21. To estimate changes in the number of high-affinity integrins as a function of the stimulating conditions, we performed extensive confocal imaging of individual cells (Fig. 7*A*) and analyzed the images in terms of their fluorescence intensity. The largest increase in fluorescent intensity, and therefore the largest enhancement in the number of high-affinity α4β1 molecules, was obtained upon co-stimulation with CXCL12 and VCAM-1-Fc (Fig. 7*B*). Stimulation with VCAM-1 or CXCL12 alone also led to an increase, albeit more modest, in activated α4β1. Surprisingly, stimulation with VCAM-1-Fc appeared to induce a higher number of high-affinity integrins than exposure to CXCL12, in apparent contradiction to the mobility data shown above.

**FIGURE 8.**
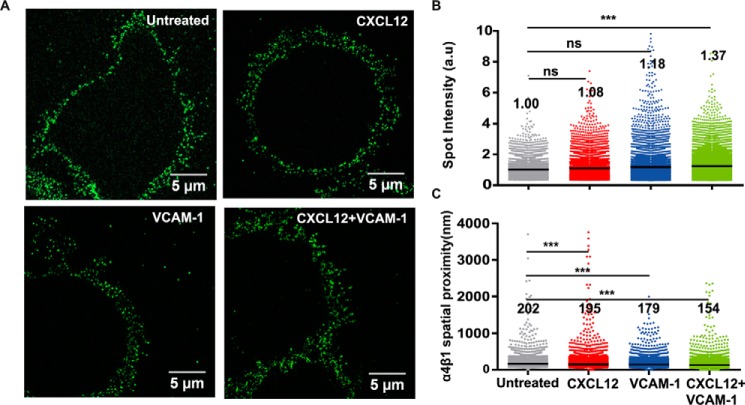
**Nanoscale organization of high-affinity α4β1integrins on T cells.** *A*, representative STED images of Molt-4 cells subjected to the indicated stimuli and stained with the HUTS-21 mAb. *B*, distribution of spot intensity per condition (at least 10 cells/condition). Values indicate the mean of the distribution. *C*, distribution of α4β1 spatial proximity on the different conditions (at least 10 cells/condition). Values indicate the mean of the distribution. *ns*, not significant (*p* > 0.05); ***, *p* < 0.001.

To then map the nanoscale organization of high-affinity integrins under different stimulation conditions, we switched to STED imaging using the HUTS-21 mAb. With an increased spatial resolution of ∼120 nm (supplemental Fig. S5), individual fluorescent spots became clearly distinguishable from the images (Fig. 8*A*). Individual spots were identified, and their intensity was quantified and normalized to the mean spot intensity obtained for untreated cells. Interestingly, very little differences in spot intensities were observed among the different conditions (Fig. 8*B*). Only co-stimulation of T cells with CXCL12 and VCAM-1-Fc resulted in a slight but significant increase in mean spot intensity, with an average number of high-affinity integrins per spot ∼1.4 times larger than on unstimulated cells. As the fluorescence intensity in each spot is proportional to the number of molecules, these results indicate no major changes in the nanoscale organization of α4β1 for the different stimulation conditions. As additional quantification, we also measured the spatial proximity between adjacent fluorescent spots. Cells stimulated with CXCL12 or VCAM-1-Fc alone showed a modest but significant reduction in their spatial proximity that became more pronounced upon co-stimulation with CXCL12 and VCAM-1-Fc compared with untreated cells (Fig. 8*C*). Taken together, these results indicate that the nanoscale organization of high-affinity α4β1 remains largely unaltered under the investigated stimulation conditions. Instead, the increased proximity of individual spots upon co-stimulation with CXCL12 and VCAM-1 suggests that newly formed high-affinity α4β1 integrins distribute on the cell membrane as independent units instead of joining pre-established integrin sites to form nano- or microclusters.

## Discussion

Using single-molecule dynamic approaches together with superresolution imaging, we explored the lateral mobility and spatial arrangement of individual α4β1integrins on T cells exposed to different stimuli that promote integrin activation, with a particular emphasis on the role of chemokines. Our data demonstrate that conditions that promote α4β1 activation, such as incubation with the chemokine CXCL12, trigger α4β1 immobilization, most probably through talin recruitment to the integrin. These results are fully in line with recent reports on other integrins, including β1 and β3 integrins associated with focal adhesions (FAs) and on αLβ2 on leukocytes, where integrin immobilization correlated with integrin activation (15–17, 31). Also, in analogy with other integrins (17, 32), robust immobilization of α4β1, triggered in our case by CXCL12 stimulation, required the tripartite interaction between α4β1, its ligand VCAM-1, and actin-binding proteins such as talin.

Although CXCL12 was able to trigger rapid immobilization and restrict the diffusion of α4β1 integrins, co-stimulation by the chemokine together with soluble VCAM-1 resulted in a 5-fold increase in immobile integrins compared with untreated cells. Although SPT approaches revealed that T cell binding to soluble VCAM-1 was capable of inducing immobilization of a small subset of α4β1, its overall effect on integrin mobility was very modest. Surprisingly, confocal and STED imaging using the activation reporter HUTS-21 anti-β1 mAb showed the induction of a larger number of high-affinity integrins upon VCAM-1 stimulation compared with CXCL12 stimulation. Together, these results suggest that, although soluble VCAM-1 is able to induce high-affinity α4β1integrins, this subset of integrins is most likely not bound to the actin cytoskeleton, therefore remaining mobile. As such, α4β1 true activation in the sense of the generation of high-affinity forms and actin cytoskeleton anchorage (through recruitment of talin) mostly occurs upon CXCL12 stimulation, which is then potentiated by co-stimulation with VCAM-1. This effect is consistent with the role of ligands in stabilizing the active form of integrins brought about by chemokine-induced transient stimulation (32).

We also performed SPT experiments of α4β1 in the presence of immobilized VCAM-1. In stark contrast to soluble ligand, immobilized VCAM-1 led to a massive immobilization of the integrin and reduction of its lateral mobility. These results can be rationalized in the context of mechanical forces being exerted between the mobile integrin and the immobilized ligand. As the integrin laterally diffuses on the cell membrane, the fixed ligand resists translation of the integrin, increasing the force and resulting in extension of the β subunit and activation of the integrin. This force-induced activation will then lead to integrin anchorage to the actin cytoskeleton and its immobilization. Addition of chemokines will further contribute to the activation of α4β1 so that the overall result is the generation of a large population of active integrins that anchor to the cytoskeleton via talin and their concomitant immobilization on the cell surface, as observed in our experiments. Such a traction force model has already been proposed for αLβ2 integrins to explain the substantially higher affinity of αLβ2 for ligand on substrates compared with the solution phase (28).

The overall conclusions of our work are based both on the well accepted role talin plays in the transmission of inside-out signaling induced by chemokines for the generation of active α4β1 and αLβ2 integrins (11, 12, 33) and on our present results achieved using a combination of different techniques to link integrin activation and integrin immobilization. Thus, our data reveal a direct correlation between rapid and transient α4β1 immobilization with talin association with β1 and induction of HUTS-21 epitopes, which constitute clear indications of integrin activation. Therefore, talin anchors the activated integrin to the actin cytoskeleton, favoring receptor immobilization. Interestingly, although the generation by CXCL12 of high-affinity α4β1 integrins and their immobilization persisted for more than 5 min, as detected with the HUTS-21 mAb, association of talin to α4β1 after chemokine stimulation was short-lived. These results might indicate that talin is only involved in bridging the first contacts between α4β1 and the actin cytoskeleton, whereas other proteins, including kindlin-3 (8), could concomitantly or subsequently cooperate with talin to stabilize the active conformation and immobilization of α4β1 over several minutes.

To facilitate T cell adhesion, crawling and diapedesis on and across the endothelium for efficient cell trafficking toward sites of inflammation, integrin-mediated adhesion (mainly involving α4β1 and αLβ2) to their respective ligands must be brief and highly dynamic, which is contributed by chemokines. This process is phenomenologically different to that of cell adhesion involving FAs, where the formation of FA clusters involving β1 and β3 integrins is required (31). Our results provide novel insights into the different biophysical properties of integrin adhesion associated with these two processes. Although, in both cases, integrin activation occurs, long-lived and robust integrin immobilization is observed on FAs as well as in the formation of large integrin-enriched microclusters (34–36). In remarkable contrast, our results indicate that physiological conditions that promote integrin activation, such as chemokines, lead to a modest but significant subset of transiently active and immobile α4β1 integrins on T cells, with no significant changes in their nanoscale spatial organization. As a low number of leukocyte integrins is involved in adhesive contacts with their ligands, their interaction is expected to be brief and highly dynamic, allowing effective T cell migration. In summary, our data indicate that fine-tuning and tight regulation of α4β1 immobilization and spatial arrangement on the cell surface are crucial processes that modulate integrin adhesiveness.

## Experimental Procedures

### 

#### 

##### Cells, Antibodies, and Reagents

The human Molt-4 T cell line was cultured in RPMI 1640 medium (Lonza, Verviers, Belgium) and 10% fetal bovine serum (Gibco). Control P3X63 and anti-β1 TS2/16 mAbs were gifts from Dr. Francisco Sánchez-Madrid (Hospital de la Princesa, Madrid, Spain), and polyclonal anti-β1A antibodies were from Dr. Guido Tarone (Turin University, Italy). The clone 18 anti-β1 and HUTS-21 anti-β1 mAbs were from BD Biosciences, and the talin clone 8D4 antibody was from Sigma-Aldrich (St. Louis, MO). CXCL12 was purchased from R&D Systems (Minneapolis, MN). Streptavidin-coated QD, biotin, and the secondary goat-anti-mouse antibody conjugated to Alexa Fluor 488 were purchased from Invitrogen. PLL and PFA were from Merck (Darmstadt, Germany).

##### Flow Cytometry

For detection of high-affinity β1 by flow cytometry, cells were stimulated for different times with CXCL12 and/or VCAM-1-Fc (R&D Systems) and fixed with 2% PFA before adding the HUTS-21 anti-β1 mAb (10 μg/ml) for 30 min at 4 °C. After washing, cells were incubated with Alexa Fluor 488-conjugated rabbit anti-mouse IgG (Jackson ImmunoResearch Laboratories). Fluorescence intensity data indicate fold-induction values relative to those from control untreated cells, which were given an arbitrary value of 1.

##### Immunoprecipitation

We essentially followed the same methodology as described previously (37). Briefly, cell lysate supernatants were incubated with antibodies, followed by coupling to protein G-Sepharose. Proteins were separated by SDS-PAGE and transferred to PVDF membranes that were sequentially incubated with primary antibodies and horseradish peroxidase-conjugated secondary antibodies. Protein visualization was achieved using Immobilon Western chemiluminescent substrate (Millipore, Billerica, MA).

##### QD-Antibody Conjugation

Streptavidin-coated QD655 was added to an equimolar solution of biotinylated clone 18 anti-β1 antibody and a 5-fold excess of free biotin to obtain a 1:1 anti-β1-QD ratio. The mixture was then gently shaken for 2 h at 4 °C, and the concentration was finally adjusted to obtain sublabeling conditions (∼0.5 μg/ml).

##### Sample Preparation for SPT

Glass coverslips (Fluorodishes, 35 mm, Menzel Glasses, Braunschweig, Germany) were coated previously with 200 μl of PLL (10 mg/ml) for 30 min at 37 °C. Molt-4 cells were diluted to 8 × 10^5^/ml in RPMI 1640 medium and spread on PLL for 30 min. Subsequently, cells were blocked by incubation for 15 min with 3% BSA, 2% human serum, and 20 mm glycine in PBS. 200 μl of the clone 18 anti-β1-QD655 conjugate was then added to the cells for 3 min at room temperature, and then cells were carefully washed 5–10 times with RPMI 1640 medium. Finally, samples were placed under an SPT microscope, and, after recording individual trajectories for 10 min, the medium was removed, and 200 μl of the different stimuli (either CXCL12 at 200 ng/ml, MnCl_2_ at 0.5 mm, soluble VCAM-1-Fc at 20 μg/ml, or combined CXCL12 and VCAM-1-Fc) were added to samples for the following 10 min.

##### VCAM-1-Fc Immobilization and Sample Preparation for SPT

100 μl of goat-human IgG F (ab́)_2_ (100 μg/ml, Jackson ImmunoResearch Laboratories) were passively absorbed onto the center of 35-mm glass coverslips by incubating for 60 min. The anti-Fc-coated area was then incubated with 100 μl of VCAM-1-Fc (20 μg/ml) for 60 min. Molt-4 cells were stretched onto immobilized VCAM-1 substrates for 30 min. Labeling of α4β1 integrins for SPT experiments and CXCL12 stimulation was performed as described above.

##### Sample Preparation for Confocal and STED Imaging

Chambered coverglasses (8 wells, Nunc Lab-TekII, Rochester, NY) were coated with 0.2 ml of PLL (10 mg/ml) for 30 min at 37 °C. Cells were resuspended in RPMI 1640 medium to a final concentration of 8 × 10^5^/ml and attached to the bottom of glasses by incubation for 30 min. Subsequently, cells were incubated for 2.5 min with the different soluble stimuli using the same concentrations as for the SPT analyses. Immediately thereafter, samples were fixed using 2% PFA and blocked for 1 h at room temperature with 3% BSA, 2% human serum, and 20 mm glycine in PBS. Next, 150 μl of the HUTS-21 mAb (5 μg/ml) was added at room temperature for 30 min, followed by secondary labeling with Alexa Fluor 488 goat anti-mouse (5 μg/ml). Finally, cells were fixed again with 2% PFA.

##### SPT Setup

Single QD655 tracking was performed using a custom setup built around an inverted microscope (IX70, Olympus) equipped with a 1.49 numerical aperture oil immersion objective (Apon ×60 total internal reflection fluorescence, Olympus). Samples were excited using a 488-nm laser (Sapphire 488–150CW CDRH) in oblique illumination configuration. A dichroic mirror (Semrock, FF500/646-Di01) was used to direct the laser light onto the sample while allowing transmission of the QD fluorescence emission, which was directed onto a CMOS camera (Hamamatsu, ORCA-Flash 4.0) after further long-pass filtering (Semrock, BLP01–635R-25). A custom-made incubator built around the microscope allowed the samples to be maintained at 37 °C with 5% CO_2_ during the measurements. During a typical experiment, movies of 1000 frames were recorded every 60 s at a frame rate of 62 Hz.

##### Single Trajectory Analyses

Analysis of individual trajectories was performed as described previously (16, 17). Briefly, a particle-tracking algorithm was programmed in MatLab to reconstruct the 2D trajectories. MSD curves were generated for each individual trajectory and subsequently fitted from the first to the fourth point to obtain the diffusion coefficient at short time lags (D_1–4_). The full distribution of D_1–4_ was then plotted as a semi-log histogram containing information from multiple trajectories on different cells. Measurements of the apparent diffusion coefficient of quantum dots on fixed cells were used to estimate the threshold value to define the immobile population. The threshold was determined as the 95th percentile of the diffusion coefficient distribution, which resulted to be D = 0.0025 μm^2^/s. The fit of the first four points of the MSD averaged over all the mobile trajectories was then used to calculate the mean diffusion coefficient. In addition, CPD analysis was performed as described earlier to quantify the diffusion of the mobile population of integrins (16, 17, 21). In essence, the CPD method calculates the distribution function for square displacements of individual molecules at different time lags. The best fitting of the square displacements distribution was obtained using a two-component model and allowed us to obtain parameters describing the diffusion and the relative contribution of two different fractions (labeled slow and fast) within the total mobile population of integrins. For both components, MSD plots were generated and anomalous exponents (α) were obtained by fitting the curves with an anomalous diffusion function: *r^2^*(*t*) = Γ*t^a^* + Δ^2^, where *r^2^* is the MSD, Γ is the transport coefficient, and Δ^2^ is the square displacement at t = 0. The slow and fast diffusion coefficients were calculated by fitting the first four points of the corresponding MSD curve using a linear model: *r^2^*(*t*) = 4D*t* + Δ^2^.

##### Confocal and STED Imaging

Both confocal and STED superresolution images of T cells were collected with a commercial microscope (CW-STED SP-5, Leica Microsystems) equipped with an oil immersion objective (HCX PL APO CS ×100.0, Leica) with 1.4 numerical aperture. Samples were excited with an argon laser at 488 nm set at 25% of its power, and their fluorescence was detected in the range of 500–580 nm. In confocal mode, images (512 × 512 pixels) were recorded with a scanning speed of 400 Hz and averaged over 3 frames with a line accumulation of 3 times. The STED laser beam intensity was set to 100% of its power (∼100–130 milliwatt), and the images (1024 × 1024) were acquired with a line accumulation of 6, a frame average of 6, and a scanning speed set at 1000 Hz.

##### Analysis of STED Images

Analysis of STED images was performed by a modified version of an algorithm described previously (38) that allowed us to automatically detect isolated fluorescent spots. Spots were fitted with a 2D Gaussian profile to determine their centroid position, peak intensity, and full width at half-maximum. Spatial proximity was quantified by determining for each spot the distance at which the nearest neighbor was located.

##### Statistical Analysis

Results are displayed as mean ± S.D. calculated from separate experiments. To determine statistical differences between the mean of different datasets, one-way analysis of variance was used, followed by Turkey's multiple comparison test (Figs. 1 and 3–6). For non-Gaussian distributed datasets, statistical differences between the means were calculated using Kruskal-Wallis test, followed by Dunn's multiple comparison test (Figs. 7*B* and 8). The resulting *p* values are indicated as follows: ns, not significant (*p* > 0.05); *, *p* < 0.05; **, *p* < 0.01; ***, *p* < 0.001.

## Author Contributions

A. S. C. and S. I. d. V. performed the experiments and data analysis. S. S. M., K. J. E. B., and C. M. contributed technical assistance to the experiments, data analysis, and interpretation. J. T. and M. F. G. P. designed and supervised the research and interpreted the data. A. S. C., J. T., and M. F. G. P. wrote the manuscript. All authors contributed significantly to the writing of the manuscript and provided useful feedback. All authors reviewed the results and approved the final version of the manuscript.

## Supplementary Material

Supplemental Data

## Footnotes

4 The abbreviations used are:

BMPC: undifferentiated human bone marrow-derived progenitor cell
SPT: single particle tracking
PLL: poly-l-lysine
MSD: mean square displacement
CPD: cumulative probability distribution
STED: stimulated emission depletion microscopy
FA: focal adhesion
QD: quantum dots
PFA: paraformaldehyde.
