# Supplementary material for: Lateral Mobility and Nanoscale Spatial Arrangement of Chemokine-activated α4β1 Integrins on T Cells
Source: J Biol Chem. 2016 Aug 1;291(40):21053–62. doi: 10.1074/jbc.M116.733709 (PMC5076515; doi:10.1074/jbc.M116.733709)
Supplement: Supplemental Data [file supp_291_40_21053__index.html]

Lateral mobility and nanoscale spatial arrangement of chemokine-activated α4β1 integrins on T cells — Lateral mobility and nanoscale spatial arrangement of chemokine-activated α4β1 integrins on T cells — Lateral Mobility and Nanoscale Spatial Arrangement of Chemokine-activated α4β1 Integrins on T Cells — Spatiotemporal Regulation of CXCL12-activated α4β1 Integrin — Supplemental Data 

# Lateral Mobility and Nanoscale Spatial Arrangement of Chemokine-activated α4β1 Integrins on T Cells

## Supplemental Data

- SPT video (.avi, 15.7 MB) - SPT video of alpha4-beta1 integrins in Molt-4 cells
- Supplementary figures (.pdf, 725 KB) - Supplementary figures S1 to S5
