# Supplementary material for: Lateral Mobility and Nanoscale Spatial Arrangement of Chemokine-activated α4β1 Integrins on T Cells
Source: J Biol Chem. 2016 Aug 1;291(40):21053–62. doi: 10.1074/jbc.M116.733709 (PMC5076515; doi:10.1074/jbc.M116.733709)
Supplement: Supplemental Data [file 10.1074_M116.733709_jbc.M116.733709-2.pdf]

Lateral mobility and nanoscale spatial arrangement of chemokine-activated α4β1 integrins on T cells

A. Sosa-Costa[1*], S. Isern de Val[2*], S. Sevilla-Movilla[2], K. J. E. Borgman[1], C. Manzo[1], J. Teixidó[2], M. F. Garcia-Parajo[1,3**]**

[1]ICFO – Institut de Ciencies Fotoniques, The Barcelona Institute of Science and Technology, 08860 Castelldefels (Barcelona), Spain.

[2]Centro de Investigaciones Biológicas (CSIC), Department of Cellular and Molecular Medicine, 28040 Madrid, Spain.

[3] ICREA-Pg. Lluís Companys 23, 08010 Barcelona, Spain

Supplemental data

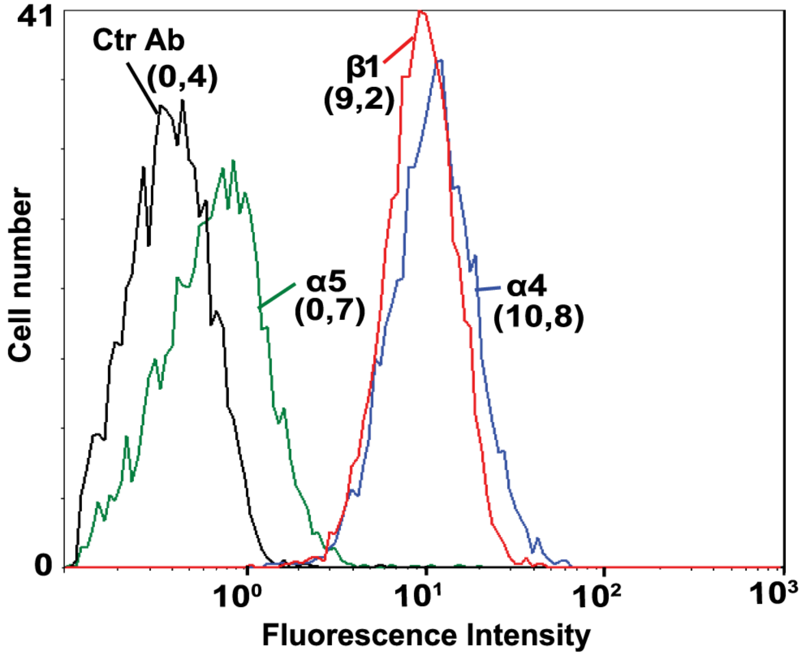

FIGURE S1. Molt-4 cells were analyzed by flow cytometry using control (black line), anti-a5 (green line), anti-α4 (blue line) or anti-β1 (red line) antibodies. Values denote mean fluorescence intensity measurements.

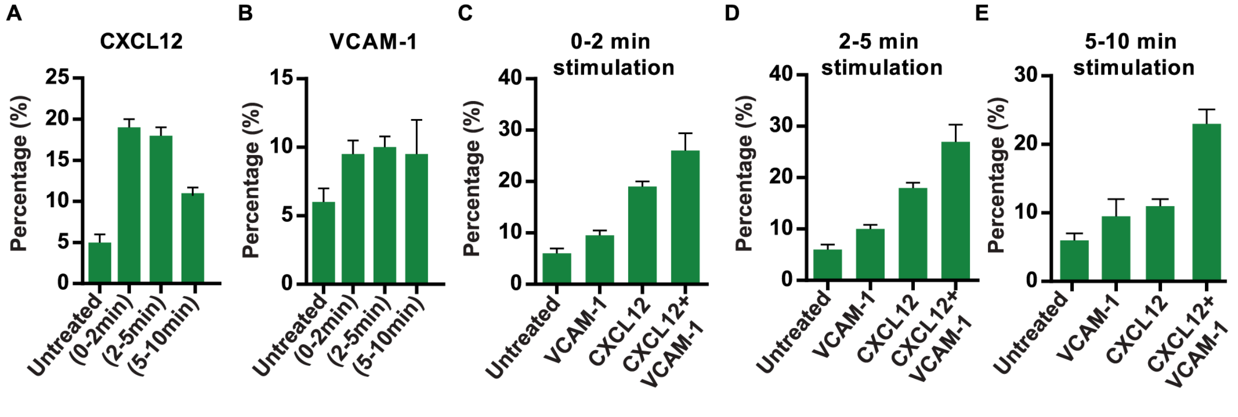

FIGURE S2: (A-E) Plots showing the percentages of immobile α4β1 trajectories for different stimulation conditions.

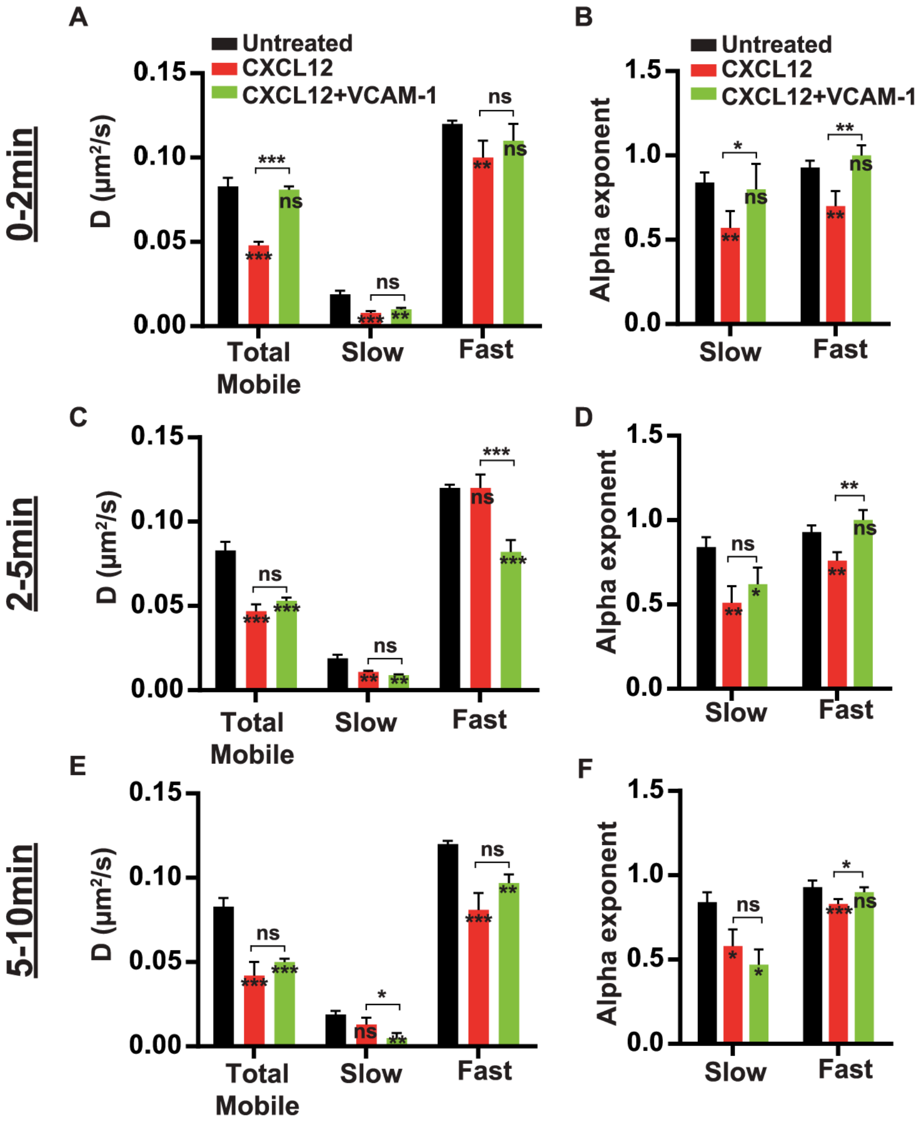

FIGURE S3. Diffusion coefficient of the total mobile trajectories, and slow and fast sub-populations (A, C, E) and alpha exponents (B, D, F) for the indicated stimulation conditions. (A, B) During 0-2 min. (C, D) During 2-5 min. (E, F) During 5-10 min.

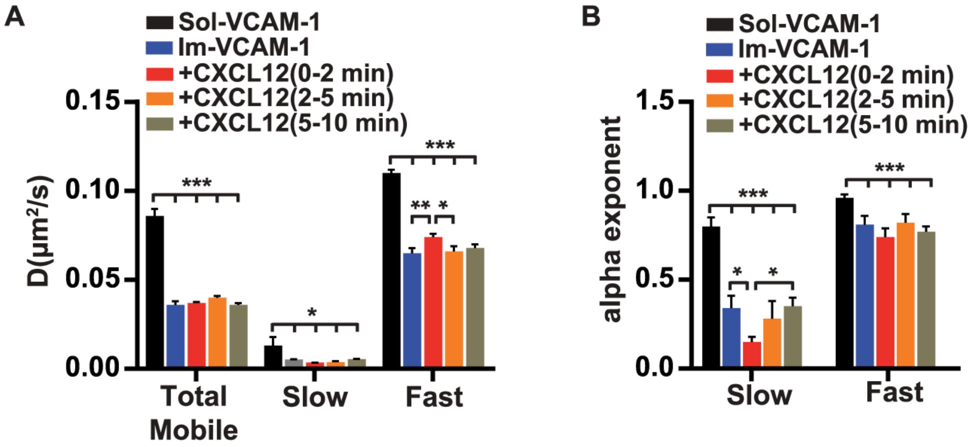

FIGURE S4. Diffusion coefficient of the total mobile trajectories as well as the slow and fast sub-populations (A) and alpha exponents (B) for the indicated stimulation conditions.

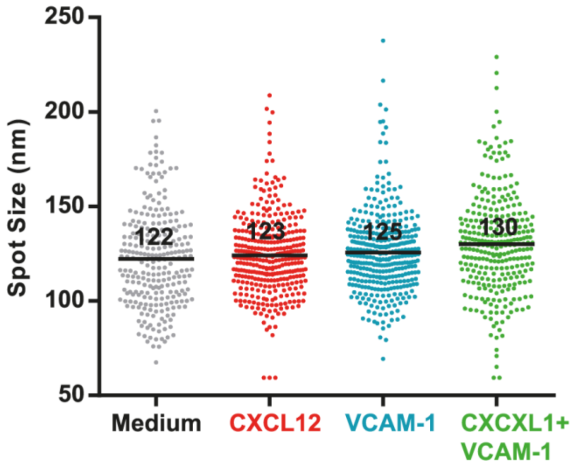

FIGURE S5. Spot size distribution was obtained by STED imaging of high-affinity integrins using the HUTS-21 mAb on Molt-4 cells incubated under the indicated conditions. Spots sizes are in all cases around 120nm, which correspond to the STED resolution under our experimental conditions. Therefore, these data do not reflect the real sizes of the fluorescent spots, indicating that if nanoclustering occurs, their sizes are well below the STED resolution and thus not directly measurable.

Movie S1. Mobility of individual α4β1 integrins on the apical side of an untreated Molt-4 cell. Representative movie of the 18 anti-β1-QD655 conjugate. Image area: 25.6×25.6 μm^2 Frame rate: 62 Hz. Length of movie: 1000 frames.
